# Impact of Early Preclinical Exposure on Academic Performance, Clinical Skills, and Confidence Among BDS Students at Private Dental College

**DOI:** 10.1155/sci5/5178600

**Published:** 2025-08-13

**Authors:** Sarah Ali, Shahzad Ahmad, Shazia Iqbal, Rakhi Issrani

**Affiliations:** ^1^Department of Dental Education and Research, HBS Medical and Dental College, Islamabad, Pakistan; ^2^Faculty of Medicine and Health Science, The University of Buckingham, Buckingham, UK; ^3^Department of Preventive Dentistry, College of Dentistry, Jouf University, Sakaka, Saudi Arabia; ^4^Department of Research Analytics, Saveetha Dental College and Hospitals, Saveetha Institute of Medical and Technical Sciences, Saveetha University, Chennai, India

**Keywords:** academic performance, BDS students, dental education, early clinical exposure, medical students, OSCE

## Abstract

**Background:** The transformation of theoretical knowledge into effective patient care is made possible by early exposure to clinical practice. It makes a substantial contribution to helping students acquire basic competencies such as empathy, problem-solving, and communication. The current study intends to determine if improved clinical interaction improves knowledge acquisition and skill acquisition, as well as to support the effect of early preclinical exposure (EPCE) on the academic performance of undergraduate dental students.

**Objectives:** To assess the effects of EPCE on academic performance, soft skills, and confidence levels among second-year BDS students at HBS Dental College, Pakistan.

**Methodology:** This comparative cross-sectional survey included a total of 150 BDS students, who were divided into two groups: Group A (EPCE) and Group B (late exposure). Academic performance was assessed using scores from the Objective Structured Clinical Examination (OSCE) and theoretical written exams. To evaluate the development of soft skills and self-confidence, both groups completed a common set of self-administered questionnaire items designed for this purpose. Descriptive statistics, including means and standard deviations, were calculated. Independent sample *t*-tests were used to compare outcomes between the two groups. A *p* value < 0.05 was considered statistically significant.

**Results:** A total of 150 students were enrolled (75 per group). Group A (early exposure) demonstrated significantly higher performance across all domains. Theory exam scores were higher in Group A (82%, SD = 3.5) than Group B (76%, SD = 4.2; *p*=0.002, Cohen's *d* = 1.55). In the OSCE, Group A outperformed in communication (85% vs. 78%, *p*=0.004, *d* = 2.15) and empathy (83% vs. 75%, *p*=0.005, *d* = 2.28). Self-reported confidence was also greater in Group A (84% vs. 77%, *p* < 0.005, *d* = 2.00).

**Conclusion:** The implementation of early preclinical placements significantly enhanced academic achievement and professional development among BDS students. Integrating early clinical exposure into the dental curriculum enriches the overall educational experience and better prepares students for the demands of real-world dental practice.

## 1. Introduction

Medical and dental education traditionally progresses from foundational preclinical coursework to hands-on clinical training. However, educational strategies are evolving as the need to integrate these phases becomes increasingly recognized. The preclinical curriculum, typically implemented during the first 2 years of undergraduate dental education, is designed to facilitate students' transition into the clinical environment [1]. Early preclinical exposure (EPCE) offers students a practical context for their theoretical knowledge, potentially enhancing their academic performance, skill acquisition, and professional development.

One of the key challenges in dental education is bridging the gap between academic learning and competent clinical practice. In traditional curricula, clinical exposure is often delayed until the later years, which may leave students underprepared for real-world patient care. To address this gap, EPCE has gained prominence across health professions' education [2]. Studies in medical education have demonstrated that EPCE contributes to increased confidence, empathy, and communication skills. However, there is a lack of robust data regarding the impact of EPCE in undergraduate dental education [3].

Most existing studies are descriptive in nature and often focus on later stages of training, with limited attention given to distinguishing between academic and soft skill development. This study aims to address that gap by evaluating whether EPCE for second-year dental students enhances not only their academic outcomes but also their soft skills and self-confidence, compared to peers who receive conventional, delayed clinical exposure.

In South Asia, there is little literature on the systematic integration of EPCE in undergraduate dental programs. The integrity of medical education in most of Asia is under threat from poor regulation, insufficient public funding, and runaway expansion of private medical schools. Competition for students' fees and an ineffective accreditation process have led to dubious admission procedures, static curricula, outdated learning techniques, and questionable assessment procedures [4]. On the other hand, worldwide, competency-based education systems are replacing traditional classroom instruction [5]. Reforms are routinely implemented by the regulatory bodies, like the nation's medical and dental health regulator, in an effort to improve the quality of education and reinforce the training process. Although such changes are difficult and time-consuming to plan and implement, curriculum reform, particularly in terms of teaching-learning and assessment methodologies, is critical, and the dental faculty must be prepared to do so [6].

In medicine and dentistry, to produce skilled and self-assured practitioners, it is crucial to bridge the gap between theoretical knowledge and practical experience. Dental students typically experience practical exposure in their final years of study. However, in order to improve student motivation and readiness, contemporary educational philosophies are moving toward the early inclusion of clinical components in the curriculum. Students can witness actual or simulated patient care through EPCE, which can greatly strengthen their comprehension of subjects taught in the classroom [7]. Similarly in dental college and hospital, where patient connection is frequent and intimate, communication skills, empathy, and clinical reasoning are essential skills for any healthcare worker. Early preclinical contact enhances these abilities while lowering anxiety related to patient involvement, according to studies in medical and dental education [8, 9] but on the other hand, the application of EPCE is not without challenges. Among some of the challenges are lack of facilities, lack of available space in health facilities, and premature interprofessional interaction taxing both teachers and students [10]. Additionally, some studies have also come with some disadvantages that are a concern of the right proportion of theoretical content provided and clinical exposure during the initial years [11]. Our hypothesis is that students' development in both the cognitive and affective domains is positively impacted by EPCE. It is thus the aim of this research to assess the effects of EPCE on academic performance, soft skills, and confidence levels among second-year BDS students at HBS Dental College, Pakistan.

## 2. Methods and Materials

This study employed a comparative quasi-experimental design with a cross-sectional survey approach, involving a total of 150 first- and second-year dental students. Participants were divided into two groups: Group A (*n* = 75), who received EPCE, and Group B (*n* = 75), who were introduced to clinical settings later in the academic year.

Group allocation was based on the existing clinical posting schedule at the institution, and students were not randomly assigned. However, efforts were made to ensure comparability between groups by examining baseline characteristics such as gender, age, socioeconomic status, and prior academic performance. No significant differences were found between the groups at baseline.

Academic performance was assessed using both theoretical exam scores and scores from the Objective Structured Clinical Examination (OSCE). The OSCE consisted of eight structured stations, each targeting core clinical competencies such as history taking, communication, and basic procedural skills. Standardized patients were employed at clinical communication stations. To reduce inter-rater variability, all examiners were trained through a structured OSCE assessor training program, and a checklist-based scoring rubric was used consistently at each station.

Soft skills and confidence were evaluated through a self-administered, standardized questionnaire, adapted from a previously validated tool developed by the Department of Medical Education. The questionnaire assessed parameters such as confidence, empathy, teamwork, and communication, using a 5-point Likert scale. A pilot test (*n* = 15) confirmed good face and content validity, and Cronbach's alpha = 0.81 indicated strong internal consistency.

### 2.1. Data Collection

Data were collected from institutional academic records, students' OSCE performance, and responses to the self-assessment questionnaire. Participants also completed a final self-evaluation of their preparedness and confidence for clinical practice. Informed consent was obtained from all participants after they were briefed about the study, and participation was entirely voluntary.

### 2.2. Statistical Analysis

Data analysis was performed using SPSS Version 24.0 (IBM Corp., Armonk, NY, USA). Descriptive statistics, including means, standard deviations, and medians, were calculated for OSCE scores, exam results, and questionnaire responses. Independent *t*-tests were used to compare performance between the two groups. A *p* value of < 0.05 was considered statistically significant.

## 3. Results

### 3.1. Academic Performance

Group A demonstrated significantly higher academic performance compared to Group B. The mean theory exam score for Group A was 82% (SD = 3.5) versus 76% (SD = 4.2) for Group B as shown in Table 1. The difference was statistically significant (*p*=0.002), with Cohen's *d* = 1.55 and a 95% confidence interval (CI) [4.75, 7.25], indicating a very large effect size.

### 3.2. OSCE Performance

In the OSCE, Group A outperformed Group B across multiple domains:

#### 3.2.1. Patient Communication Skills

Group A scored 85% (SD = 3.0), while Group B scored 78% (SD = 3.5). This difference was statistically significant (*p*=0.004, Cohen's *d* = 2.15, 95% CI [5.95, 8.05]).

#### 3.2.2. Empathy

Group A achieved a mean score of 83% (SD = 3.2) compared to 75% (SD = 3.8) in Group B (*p*=0.005, Cohen's *d* = 2.28, 95% CI [6.87, 9.13]).

Table 2 shows the OSCE communication and empathy score comparison between groups.

### 3.3. Self-Reported Confidence

Students in Group A reported significantly higher confidence levels, with a mean of 84% (SD = 3.4) compared to 77% (SD = 3.6) in Group B. The difference was statistically significant (*p* < 0.005, Cohen's *d* = 2.00, 95% CI [5.87, 8.13]), as shown in Table 3.

Thus, overall, Group A regularly delivered better results in terms of academic achievement, OSCE scores, and confidence levels, as shown in Table 4. This implies that early preclinical experience may be crucial in determining dental students' professional and interpersonal skills as well as cognitive outcomes.

## 4. Discussion

### 4.1. Academic Achievement and Learning Experience

According to the study's findings, academic performance is considerably improved by EPCE. When it came to written tests and OSCEs, students in Group A performed noticeably better than those in Group B. The findings are consistent with earlier research showing that early clinical exposure improves knowledge retention and application by helping students understand theoretical topics contextually [12, 13]. A similar comprehensive evaluation confirmed that learning higher ideas in a clinical setting is better understood and retained when it is practical and case-based [14]. Students with a clinical orientation achieve noticeably better outcomes than those with less clinical experience; this study also showed a substantial difference in the academic performance of the students who attended the clinical early. Through its ability to connect the fundamental sciences with practical clinical applications, EPCE promotes deeper learning [15].

### 4.2. Enhancement of Interpersonal Skills

The belief that directs patient involvement, as opposed to merely theoretical training, is a more effective way for noncognitive abilities to develop and is supported by higher communication and empathy scores among students with EPCE. Early patient contact develops interpersonal and sympathetic abilities, which are critical for dentistry and medical professionals, according to previous research [16]. Early patient involvement ensures that communication and empathy, which are basic parts of patient care, are established. Effective communication skills are essential for positive patient–doctor interactions in the healthcare, which leads to better patient management and satisfaction outcomes [13, 14]. This is consistent with the OSCE results from this study, which showed that Group A participants did marginally better than Group B participants in patient communication and empathy. Students with a clinical focus have noticeably higher outcomes than those with less clinical experience. It is not enough to consider clinical exposure alone in terms of developing clinical skills but should be able to work as a team. Students need years of consolidation in clinical practice settings before they may be considered clinically competent. Another study supports that student must develop the interpersonal and teamwork skills necessary to work in the interdisciplinary teams that define the contemporary healthcare environment [15].

### 4.3. Improved Student Confidence

The results of this study showed that 90% of students in Group A reported feeling very confident in handling clinical situations, while only 70% in Group B. This is consistent with previous research that highlights the beneficial effects of early clinical experiences on students' confidence levels. The rise in self-reported confidence among students is consistent with previous research that demonstrates that frequent early clinical engagement lowers anxiety and increases professional assurance through familiarity with the experience [17]. According to Dornan et al., this kind of experiential learning strengthens learners' self-efficacy by encouraging them to achieve competency through situational practice and creating a sense of belonging in clinical settings. These varying perspectives reinforce the importance of EPCE in shaping both students' academic performance and their psychological readiness for clinical practice. They also provide a theoretical foundation for interpreting the findings of the present study.

### 4.4. Potential Bias and Other Theories

Even though positive findings linked to EPCE are supportive, it is important to interpret the results cautiously because there may be other possible causes. Self-selection bias is a serious concern since students who are naturally more driven, capable, or self-assured may gain more from EPCE or may have been more likely to achieve regardless of the sort of exposure. Due to their innate qualities rather than just EPCE, these students might participate more actively, benefit more from clinical situations, and achieve better. It is challenging to eradicate this kind of bias without appropriate randomization, which restricts the ability to account for baseline differences across groups and compromises the study's internal validity [18]. Since there is no random assignment, we cannot be positive that EPCE, not these unmeasured confounding factors, is the cause of the gains in student outcomes. Similar to this, earlier research in medical education has emphasized how student attributes, such as self-efficacy and past experience, affect performance, indicating the necessity of strict control designs when assessing educational interventions [19, 20].

### 4.5. Limitations and Risks of EPCE

Although EPCE has numerous educational benefits, it also has drawbacks. Clinical settings can overwhelm students in the early phases of their medical or dental education, especially while their core knowledge is still growing. This is a major worry. Instead, then improving learning, early exposure could cause confusion, anxiety, and disengagement if basic science and communication principles are not sufficiently understood. Literature highlighting that ill-timed or insufficiently supported clinical contacts might have detrimental consequences on inexperienced learners supports this concern [12]. Moreover, the quality and equality of the clinical learning experience may be jeopardized by the logistical difficulties that EPCE programs frequently encounter, such as a greater workload for faculty, a lower capacity for supervision, and irregular patient availability [21]. EPCE may not provide the desired results or may even deter students if its structure is poorly thought out, lacking clear objectives, appropriate teacher training, and sufficient student support.

On the other hand, EPCE can be very useful for students' awareness in community-based programs, pre-clinical and clinical set-ups depending on the context of its efficacy and durability by its careful and methodical implementation [22]. Thus, our findings corroborate those of other recent research on clinical experience as a vital part of medical and dental students' education. The conventional concept of patient interaction correlates with course knowledge and enhances the practical experience of aspiring graduates. Comparing the identified outcomes of this study to the earlier studies and pointing out the points are explored in this section. According to a number of academics, the initial clinical encounter helps students do better academically and prepare for the workforce.

## 5. Limitations

This study has limited sample size; a potential source of selection bias is the fact that students were not randomly assigned to early and late exposure groups. Second, because the study was limited to a single dental institution, its findings may not be applicable to other educational contexts with diverse resources or curricular structures. Third, self-reported questionnaires may have incorporated social desirability bias and may not correctly reflect students' real clinical competencies when used to gauge confidence and soft skills. Last but not least, the study solely evaluated immediate results and lacked qualitative information that would have shed light on the students' experiences in more detail.

## 6. Conclusion

According to this study, undergraduate dentistry students' academic performance, soft skills, and confidence are all markedly improved by EPCE. Prior exposure to clinical settings was associated with much higher confidence in patient interactions and better performance on OSCE evaluations and theoretical exams. These results provide credence to the idea that including structured EPCE programs within preclinical curricula is a smart way to close the theory-practice divide. In addition to contributing to the existing body of evidence, the findings of this study have important implications for curriculum development and educational policy in dental education. EPCE should be institutionalized into dental education frameworks to encourage experiential learning from the start, according to policymakers and academic leaders. Integrating EPCE into the curriculum as a whole can help students become more prepared, enhance patient-centered abilities, and match learning objectives with the needs of the healthcare industry.

## 7. Future Recommendations

To reduce bias and support causal inferences, randomized controlled designs should be used in future studies. The findings would be more broadly applicable if multicenter studies were conducted in a variety of educational environments. Furthermore, using qualitative approaches such as in-depth interviews, reflective journals, and focus groups may provide a deeper understanding of students' viewpoints and experiences. The long-term effects of early clinical exposure on students' clinical competency, empathy development, and preparedness for independent practice should be evaluated through longitudinal research. Lastly, to better guide curriculum design and policy development, future research should examine the varied impact of EPCE across different student demographics and account for any confounding factors.

## Figures and Tables

**Table 1:** Comparison of academic performance between early and late clinical exposure groups.

Group	Mean score (%)	Standard deviation (SD)	*p* value
Group A	82	3.5	0.002
Group B	76	4.2	

**Table 2:** OSCE communication and empathy score comparison between groups.

Domain	Group A mean (%)	SD	Group B mean (%)	SD	*p* value	Cohen's d	95% CI
Communication	85	3.0	78	3.5	0.004	2.15	[5.95, 8.05]
Empathy	83	3.2	75	3.8	0.005	2.28	[6.87, 9.13]

**Table 3:** Self-reported confidence levels between early and late exposure groups.

Group	Mean confidence score (%)	SD
Group A	84	3.4
Group B	77	3.6
Not confident	2	10

**Table 4:** Summary of findings (in percentage %).

Outcome	Group A (%)	Group B (%)	Difference (%)
Improved academic scores	92	80	12
Enhanced OSCE skills	85	78	7
Increased confidence	90	70	20

## Data Availability

The data set used in the current study will be made available on request from the corresponding author.
